# Supplementary material for: Chromothripsis during telomere crisis is independent of NHEJ, and consistent with a replicative origin
Source: Genome Res. 2019 May;29(5):737–49. doi: 10.1101/gr.240705.118 (PMC6499312; doi:10.1101/gr.240705.118)
Supplement: Supplemental Material [file supp_gr.240705.118_Supplemental_file_1.zip › contigs/annotated_contigs/DB51/contig.2.DB51_length_655_mean_cov_5.98167938931.docx]

**DB51_length_655_mean_cov_5.98167938931**

```
TTTTAATAATGCATAAGGGGAATTTTTAGGAGGTTCTTTTATTACAAAAGAAATACAGTATATCCTCAGGGTAAAAATATTCAAGTATT
 >chr14:67595770-67595949 - E=6e-96 p=1e-02
TCAGAAATATTAAAAAAAAAAAGTAGAAGTGGCTGGGCACGATGGCTCACACCTTTTATCCCAGCACTTTGGGAGGCCAAGGCAGGTG|

GA|GCACGTAAGCACCCAACTGAGGACTGCATGATGCTGGGGCTTGAGAGAATAAAAAAGAAAAAGTGAAGAGCATAATAGTAGCTAGG
>chr14:68052675-68053125 - E=5e-244
CCAGAGTTAGTTCCCTCCAAATATGTCCCTTTACCTACTAGCTGAACATTGATACGGAGATGCAACACCACCTGAACCTTTGGAATACT

AATTACAGACAAGACCAACAATTTTATCTGGGCAGGGCCCTTGCAGAGCACTAGCATAACCCCAGTTTCCTTTCTAAGCTCCAAGCCCA

CCACATCTTGCCAGAGTAGAACGTTTTAACAGCTGTCAGACCTACCTTGGGAGCAGCCATCCGGAAGATCAACTTCTCAATGTGGCTTT

TTCCAGAGCTCTTGTCTGGGATAGATCTAATCACAAGCATGAAGGCATCCCTGCAGCCACAAAACGTGGTCACTGAAGAGGAGTGGTAA

GTGATG|GGGAGTTGGTGGGGGAAGGAAGAAGTCG
```
